# “This needs to be told to everyone”: Content analysis of written immediate responses from an online experiment examining health warning messages about alcohol consumption and breast cancer risk

**DOI:** 10.1371/journal.pone.0338687

**Published:** 2025-12-12

**Authors:** Allison Anbari, Zachary Massey, Abigail Adediran, Na Wang, LaRissa Lawrie, Priscilla Martinez, Denis McCarthy

**Affiliations:** 1 Sinclair School of Nursing, University of Missouri, Columbia, Missouri, United States of America; 2 TSET Health Promotion Research Center, Stephenson Cancer Center, Department of Health Promotion Sciences, Hudson College of Public Health, University of Oklahoma Health Sciences, Oklahoma City, United States of America; 3 Department of Communication, University of Missouri, Columbia, Missouri, United States of America; 4 School of Journalism, University of Missouri, Columbia, Missouri, United States of America; 5 Alcohol Research Group/Public Health Institute, Emeryville, California, United States of America; 6 Department of Psychological Sciences, University of Missouri, Columbia, Missouri, United States of America; SUNY Downstate Health Sciences University, UNITED STATES OF AMERICA

## Abstract

Alcohol consumption increases breast cancer risk. We evaluated the responses of 748 United States female participants ages 21–29 to health warning messages addressing the relationship between alcohol consumption and increased breast cancer risk. In an online experiment, participants were randomly assigned to view standalone health warning messages about alcohol, breast cancer, and breast cancer health effects with varying picture and text attributes. Participants then completed post-message exposure assessments that included an immediate open-ended response to the message prompt. We conducted a qualitative content analysis of the responses and coded deductively based on constructs from the Message Impact Framework including message reactions, attitudes and beliefs, and behavioral intentions. These constructs and corresponding variables were present in participants’ responses. Response type did not vary by participants’ demographics or the attributes of the health warning message they viewed. The code *new information* was applied to 20% of the responses, indicating that those participants had no prior knowledge of alcohol and breast cancer risk. Alcohol and breast cancer messaging could impact drinking behaviors. Given the frequency of responses indicating a lack of awareness, more work in cancer prevention and population health messaging is warranted.

## Introduction

### Breast cancer and alcohol

One in eight women in the United States (US) will be diagnosed with invasive breast cancer in their lifetime [1]. Current estimates suggest that 40% of breast cancers may be preventable [2]. One modifiable risk factor is alcohol consumption [3]. Alcohol is a Group I carcinogen [4,5], known to increase risk for breast cancer by increasing endogenous estrogen levels in pre-menopausal females. Estrogen levels are linked to higher breast density and breast cancer development [6–9], and as such, this is one of the biological mechanisms of alcohol’s effect on breast cancer risk. Importantly, the relationship between alcohol and breast cancer risk is dose-dependent with no lower threshold – meaning all levels of alcohol consumption increase breast cancer risk, and risk increases as consumption increases [3,4,10,11]. Pre-menopausal breast cancer risk increases by as much as 34% with one and a half drinks per day [3,10].

Recent cancer statistics indicate that the cancer burden for women has increased. The American Cancer Society estimates that, in the US, younger females have an 82% higher incidence of cancer than males, which is a significant increase from the incidence of 51% in 2002 [12]. This increase is concerning, especially in view of evidence demonstrating that the relationship between alcohol consumption and breast cancer risk is not well known publicly [13–16] and that breast cancer risk is frequently underestimated [17]. Lack of awareness and underestimation of the risk are problematic public health concerns, given that alcohol consumption remains prevalent [18]. While a recent Gallup Poll reported a decline in the prevalence of alcohol use among US adults to 54%, this is consistent with data on the prevalence of past 30-day alcohol consumption from the recent editions of the National Survey on Drug Use and Health [19,20]. Further, findings from a National Alcohol Survey analysis that compared 2019 and 2024 data show an increase in the volume of alcohol consumed among past-year drinkers, suggesting current drinkers are increasing their alcohol use [21]. This finding is particularly relevant to the cancer risk from alcohol given the dose dependency of the risk. Therefore, effective health communication is warranted to increase awareness and address this public health concern.

In January 2025, the US Surgeon General issued an advisory report recommending that health warning labels about cancer and alcohol be included on alcohol packaging [22]. Current US alcohol packages and product warnings do not include nor are they required to include information about cancer risk. Alcohol product warnings in the US remain unchanged since 1988 and use warning themes such as risks related to alcohol consumption during pregnancy, dangers related to operating heavy machinery, and a general statement that alcohol consumption may cause health problems [23]. The themes are not generally relevant, nor do they include potential consequences from the health harms (e.g., in utero effects or long-term injury/death). Other countries, such as South Korea and Canada [24–26], are making concerted efforts to inform the public about the carcinogenic effects of alcohol. Ireland was set to be the first country to require the inclusion of labels about the relationship directly on alcohol products, starting in May 2026; however the mandate has been delayed [27–29].

Health communication and evidence-based health warning messages (HWMs) are crucial for cancer control strategies [30]. Health warning messages that include pictures about tobacco health harms, such as cancer, have been studied and proven successful at reducing the appeal of tobacco products and intentions to use tobacco [31,32]. However, less is known about what type of HWMs regarding cancer risk and alcohol consumption would be the most effective in achieving a reduction in the appeal and intentions to consume alcohol. To date, the few studies about HWM attributes and their effectiveness and thus their potential use for US alcohol product labels have mostly focused on colon, liver, or oral cancers using narrative messages compared to pictorial messages. Narrative health warning messages are defined as messages that include pictures and/or words about people with a lived experience (e.g., a person with a feeding tube as a result of cancer). Pictorial messages, or the non-narrative health warnings in these studies, included graphic images of health consequences (e.g., pictures of cancerous lesions) [33–36]. Other research has examined breast cancer or cancer messaging (text and/or icons) alongside other alcohol health harms [37–39]. Our online experiment and the results we report here are among the first in the US to examine HWMs specifically and solely about alcohol consumption and breast cancer risk [40].

Understanding the potential impact of HWMs addressing alcohol and breast cancer risk is important because cross-sectional survey studies in other countries have demonstrated that support of alcohol control policies increases when awareness of the relationship between alcohol consumption and cancer risk increases [14,18,41,42]. Likewise, in an online experimental study in the United Kingdom, young adults’ interest in alcohol products was shown to decrease after they viewed alcohol health warnings on alcohol packages (including warnings about cancer risk) [43].

### Evaluating health warning messages

Before HWMs can be placed on alcohol products, or disseminated in campaigns, they should be tested among the intended audience [31,44]. Testing involves understanding the format and content of the warnings as well as which message attributes elicit reactions, influence attitudes or beliefs, or indicate potential for behavior change [31]. One method of assessing HWMs is exposing participants to hypothetical HWM designs and evaluating their perceptions and reactions via spontaneous reactions provided in open-ended response opportunities. Including open-ended response opportunities or thought-listing tasks alongside closed-ended survey items is an often-used method to evaluate attitudes of participants that may not have been otherwise assessed [45,46]. Open-ended response opportunities allow participants to freely express their immediate perceptions about health warnings, often providing rich insights that may not be captured in survey items on their own terms, without the confines of the researchers’ closed-ended items [45–48]. No studies, to our knowledge, have assessed HWM reactions and perceptions within open-ended and spontaneous responses to HWMs about alcohol consumption and increased breast cancer risk.

### The message impact framework

This study was informed by the Message Impact Framework, derived from meta-analyses of tobacco health warning research [44,49]. The Message Impact Framework identifies constructs “sets” that are theorized as antecedents of behavior change. The framework posits that message attributes influence individual message reactions, such as attention to the warnings and emotional reactions. Message reactions are posited to impact attitudes and beliefs, which include participants’ perceptions about the severity of the harm, their susceptibility to the harm, and their efficacy to respond to the health harm. Lastly, attitudes and beliefs are posited to influence behavioral intentions and health behaviors such as reducing or stopping alcohol consumption [44,49]. For this study, we used content analysis techniques to code the open-ended spontaneous responses to HWMs guided by the Message Impact Framework.

### Objectives

The open-ended responses (hereafter referred to as responses) were collected during an experimental study evaluating different alcohol and breast cancer HWM designs [40]. Here, we report qualitative directed content analysis and findings, the corresponding quantitative analysis, and results not reported elsewhere. Coding was guided by the variable sets within the Message Impact Framework, which were used for directed content analysis of the responses [50–53]. Our goal was to answer the following questions:

1) What components of a) message reactions, b) attitudes and beliefs, and c) behavioral intentions to reduce or stop drinking alcohol were present in participants’ responses after health warning message exposure?
2) When considering the type of health warning message (text-only or picture-and-text) and the health harm that participants were exposed to, were there similarities or differences in: a) message reactions, b) attitudes and beliefs, and c) behavioral intentions to reduce or stop drinking alcohol present in participants’ responses?
3) When considering the health harm participants were exposed to and select demographics (e.g., age or education), were there similarities or differences in: a) message reactions, b) attitudes and beliefs, and c) behavioral intentions to reduce or stop drinking alcohol present in participants’ responses?

## Materials and methods

### Participants

This study analyzed data from an online experimental study evaluating different alcohol and breast cancer HWM designs and attributes [40]. We evaluated the perceptions of young adult female participants in the US who self-reported alcohol consumption in the last 30 days after exposure to text-only or picture-and-text HWMs about how alcohol consumption can increase risk for breast cancer (described below). Data collection occurred from April 1 to April 24, 2024, and was facilitated by the Qualtrics social marketing research firm. Qualtrics recruited and compensated participants from a panel, and the research team designed all experimental materials. Sample quotas were approximated using US Census data and estimates of the national population of females who drink [54,55]. The University of Missouri Institutional Review Board approved this study (2099603). Our reporting here is guided by the Consolidated Criteria for Reporting Qualitative Research Checklist (COREQ) [56] and content analysis reporting guidelines [53].

### Procedure and design

In an online experiment, participants provided informed consent before completing baseline demographic and substance use questions. Participants were randomly assigned to view one standalone HWM (text-only or picture-and-text) about the relationship between alcohol consumption and breast cancer risk along with a description of one of three breast cancer health harms (mastectomy, hair loss from chemotherapy, and mortality). After exposure to the HWMs, participants were presented with an open-ended thought-listing task eliciting their immediate response to the HWM. Outcome measures, guided by the Message Impact Framework variable groups, were then assessed using additional survey items. The end of the survey included a debrief about the research and contact information for the Substance Abuse and Mental Health Services Administration for resources about alcohol consumption and a website link to healthywomen.org for resources about breast health.

### Experimental stimuli – health warning messages

We designed HWMs to include the four components theorized for effective warnings [30]: a signal word (i.e., “Warning!”), identification of the risk (i.e., the connection between alcohol consumption and breast cancer risk) description of the effect (i.e., a single health harm resulting from cancer (plus or minus a picture depicting the harm), and steps that could help avoid the risk (i.e., a self-efficacy statement about alcohol consumption reduction and speaking with a healthcare provider about decreasing risk). The HWMs appeared on the screen alone and all text was the same font size, white, and placed on a black background. The picture-and-text warnings were formatted with the same sized graphic images based on previous research testing tobacco health warning designs [57,58]. All HWMs were formatted to match in number of words, reading level, and design. In our pilot with a national sample, draft HWMs using the topics of mortality, hair loss from chemotherapy, lymphedema, and mortality were tested. The health harms with the highest mean scores for perceived message effectiveness, mastectomy, mortality, and hair loss from chemotherapy, were used for our main experiment. Additional description, examples of the HWMs, and full results of the experiment, including the quantitative assessment of the variable groups from the Message Impact Framework, are reported elsewhere [40].

### Measures

#### Demographic measures.

Prior to exposure to the HWMs and the open-ended response opportunity, participants completed standard demographic assessments that included items related to age, race (grouped as White, non-White); ethnicity (Hispanic, Latina, or Spanish origin); education (grouped as high school or less, some college or above), annual household income (less than $50,000, $50,000 to $100,000, or more than $100,000), and health status (1 = poor to 5 = excellent).

### Alcohol use status

Alcohol use status was measured using alcohol consumption frequency with an item asking, “*How many times have you had at least one drink of alcohol in the past 30 days*?” followed by a prompt to select from 1 to 30 times.

### Open-ended immediate response

The unit of analysis for this study was the response provided by participants who viewed the HWMs with health harms about mastectomy, hair loss from chemotherapy, and mortality. Participants encountered the response opportunity before completing the remaining survey items related to the Message Impact Framework variable sets. Participants were prompted to: *“Please type in every thought that came to mind while looking at the messages. Don’t worry about spelling or punctuation. Please list as many thoughts as possible that you can recall thinking while looking at the messages.”* [59]. The prompt was shown on the same survey screen as its corresponding textbox where free text entry was enabled without word count limits. Participants needed to enter content (even blank spaces) into this area before proceeding with the rest of the survey.

### Data preparation

We excluded 21 blank/unusable responses from the HWM health harms (responses such as “*I’m*” or “*gut*”). This resulted in a total of 748 responses used for analysis. The number of participants who viewed the different types of messages (i.e., text-only or picture-and-text and health harms) is presented in Table 3. Chi-square tests showed experimental assignment to health harm and HWM type (text-only or picture-and-text) and demographic characteristics remained balanced after data preparation [40].

### Approach to analysis

Data were analyzed in three steps. Step 1 included assessing the responses to identify the constructs from the Message Impact Framework using directed content analysis [50–52]. Step 2 included assessing the association between response codes and message types (text-only or picture-and-text) and health harms (mastectomy, hair loss from chemotherapy, and mortality), respectively. Step 3 included assessing the moderating effects of the associations between demographic traits and response codes. All the qualitative analysis was done using Dedoose software, while quantitative analyses were done using SPSS software.

### Step 1 directed content analysis

Use of directed content analysis provided a systematic approach to qualitative analysis given the high number of open-ended responses collected as part of a larger quantitative experimental study [53]. In Dedoose, each response was labeled with a code and participant descriptors (i.e., age, race, ethnicity, education, income, health status, and drinking history, and drinking history).

### Initial codebook creation and structure

The research team deductively created the codebook before coding was initiated and inductively updated it during analysis as additional patterns in the responses were identified [50–52]. Thus, the majority of our code labels were created using the constructs and operationalizations from the Message Impact Framework [44] (See Table 1). These included the overarching categories (parent code labels) based on the theoretical constructs of the Message Impact Framework, including *message reactions*, *attitudes/beliefs*, and *behavioral intentions,* as well as subcategories of corresponding variables within each of these sets. Example subcategories included *attention* for *message reactions* and *stop drinking* for *behavioral intentions*. Within the codebook, we documented key words and phrases that related to the parent code labels and corresponding variables as subcategories. The definitions and list of keywords evolved as coding progressed, however initial keywords/definitions always served as guideposts or coding rules [52].

**Table 1:** Codebook and Danalysis as described by Hsieh definitions.

Code	Definition	Key words
**Nonsubstantive**	Participants did not offer any substantive response.	IDK/None/No/Don’t know
**Message Reactions**		
Fear/Anxiety/Worry	Participants interpret the message as threatening, scary, or worrisome.	fear, scared, worried, this is scary, I am worried
Cognitive Elaboration	Participants provide further interpretation and meaning to the message, reflecting on topics related to its content.	expansion of the health warning but not about risk, tangential application of the health warning content, this made me think about…., Alcohol can cause other….
Attention	Participants demonstrate attentiveness to and recall of the message.	discrete restatement of the health warning message without mention of impression/feeling/elaboration; Alcohol increases the risk for breast cancer, drinking alcohol can cause breast cancer
Hope	Participants appraise the message as consistent with their desired future outcomes.	hope, encouraged, motivation, I am hopeful because drinking less can decrease risk
Sad	Participants interpret the message as sad.	sad, sadness, guilt, disappointed, this is sad
Shock/Surprise	Participants interpret the message as shocking.	shock, surprise, wow, alarmed, I was surprised when reading the messages
Seeking Information	Participants express a need for additional resources.	data, research, source, science, truth, asks questions, I wondered what the source of this was
Survey Specific	Participants provide information about the stimuli and survey design themselves, such as mentions of wording, colors, pictures, etc.	specific mention of message design, text, pictures
**Attitudes and Beliefs**		
First person and/or Perceived Efficacy	Participants express the belief that they can prevent or control the risk of breast cancer.	recognition of the threat to themselves (uses first person) and whether they are at risk (or not), I am at risk, I am already at risk, I don’t drink enough to be at risk
Dismissal of current threat	Participants specifically express the belief that they are not at risk of breast cancer.	mentions that they are not a risk right now or ever because they don’t drink enough, messages don’t apply to them
Others – Perceived Threat	Participants express the perceived risk of alcohol-related breast cancer harm and the magnitude of that breast cancer harm caused by alcohol.	recognition of the threat to others, threat deflected generalized threat, mentions risk but applied to others, People who drink a lot are at risk
Thinking about family/friends	Participants specifically express concerns about their family’s or friends’ risk of alcohol-related cancer harm.	mentions family members, mothers, children, friends
New Information	Participants report that this is new information or that she was not aware of it before.	didn’t know this, this is news, this is new information, I wasn’t aware
**Behavioral Intentions**		
Reduce drinking	Participants desire to reduce consumption.	cut back, slow down, I should think about drinking less, I will drink less
Stop drinking	Participants desire to stop consumption.	stop, quit, makes me want to quit drinking, I will quit drinking

We initiated directed content analysis as described by Hsieh and Shannon (2005) using the predefined code labels within the codebook. Each response was coded independently and according to the codebook by a minimum of two research team members who were also involved in study design, data preparation, and the development of the codebook. Responses that warranted additional code labels that were not listed in the initial codebook were flagged and discussed (e.g., responses to be labeled later as *sad*, added as a subcategory of the parent code *message reactions*). Interim results and discrepancies were discussed during analysis-specific team meetings. We maintained an audit trail within the codebook as code label definitions evolved or required clarifications/discussion [51]. Parent and subcategory code labels were collapsed when discerning between two categories or corresponding Message Impact Framework variables became less discrete or more complex. For example, the code labels related to the variables of *perceived susceptibility* and *perceived threat* were adjusted to *perceived threat* and *first person and/or perceived efficacy*. Similarly, we added subcategories to *message reactions* such as *sad* and *shock or surprise* when the team noticed their frequency. Table 1 is the final codebook.

Verification of the appropriateness of code labels and their application to responses was ongoing as the research team reviewed each flagged response to reach consensus. To validate rigor and consistency, we viewed responses in groups by code label, confirming reliability and identifying patterns of language within the grouped responses. Patterns of language contributed to objectivity and trustworthiness as the research team verified that the responses were grouped appropriately by code label. We then assessed whether a certain element or variable set of the Message Impact Framework appeared more or less frequently in certain participants’ responses.

### Step 2 Chi-square tests of independence

Once each response was labeled, we were able to group responses by Message Impact Framework constructs and subcategories. For example, we identified the number of responses that were labeled with the *attention* subcategory of *message reactions*. This allowed us to the code labels (i.e., response types such as *attention*) as dependent categorical variables and conduct analysis to answer research question 2, to examine whether the message types (text-only or picture-and-text) were associated with participants’ response types (e.g., *attention*). We analyzed the four parent code labels (message reactions, attitudes and beliefs, behavioral intentions, and new information) and their highest frequency subcodes (attention, first person and/or perceived efficacy, and stop drinking). We used SPSS to conduct Chi-square tests of independence.

### Step 3 Moderation analysis

To address research question 3, we used moderation analyses (ANOVA and regression) to test whether demographic characteristics, including age, race, ethnicity, education, income, health status, and drinking frequency, would have a moderating effect on participants’ response types (again analyzed as the dependent categorical variable). For race and ethnicity, a categorical variable, we used two-way factorial ANOVA to examine whether the effect of health harm HWM on response type (e.g., responses labeled with *attention*) was moderated by race and ethnicity respectively. For age, income, education, health status, and drinking frequency, which were treated as dummy coded continuous variables, we used hierarchical multiple linear regression to test moderation effects. For the analysis for research questions 2 and 3, all statistical significance was evaluated at the 0.01 level to minimize the risk of Type I error.

### Sample and dataset characteristics

Table 2 shows all participant characteristics. We included open-ended, substantive responses from 748 female young adult participants between 21 and 29 years of age (*M *= 25.3, *SD = *2.6) who reported consuming alcohol in the past 30-days and viewed the health harm HWMs. Most participants identified as White (71.7%), followed by Black or African American (11.5%), Asian (4.5%), American Indian or Alaskan Native (2.0%), Native Hawaiian or Other Pacific Islander (0.4%), more than 1 race (4.8%), or another race not listed (5.1%). Most did not identify as Hispanic, Latina, Latinx, or of Spanish origin (81.8%). In terms of education, 26.7% of participants were high school graduates, while 24.7% had some college education, followed by bachelor’s degree (19.1%), associate’s degree (10.6%), master’s degree (9.4%), less than high school (5.7%), and professional or doctoral degree (3.7%). 35.8% of participants reported their annual household income as less than $50,000, while 35.0% of people reported it between $50,000 and $100,00. Most participants perceived their health as good (43.2%)

**Table 2:** Sample characteristics overall and by health harm HWM. All participants (n = 748) reported currently drinking alcohol.

		HWM Condition		
	Overall (*N* = 748) Unweighted %	Mortality condition (*n* = 245) Unweighted %	Mastectomy condition (*n* = 253) Unweighted %	Hair loss condition (*n* = 250) Unweighted %
**Age**				
Mean (*SD*)	25.3 (2.6)	25.3 (2.6)	25.3 (2.6)	25.4 (2.6)
**Racial identification**				
White	71.7	71.8	71.1	71.2
Black or African American	11.5	11.0	13.0	10.4
Asian	4.5	4.9	4.0	4.8
American Indian or Alaskan Native	2.0	2.9	0.8	2.4
Native Hawaiian or Other Pacific Islander	0.4	0.4	0.8	0.0
More than one race	4.8	5.3	4.7	4.4
Other	5.1	3.7	4.7	6.8
**Hispanic, Latino, Latina,** **Latinx, or Spanish origin?**				
Yes	18.2	19.2	15.8	19.6
**Education**				
Less than high school	5.7	5.3	6.7	5.2
High school graduate or GED	26.7	31.0	23.3	26.0
Some college	24.7	21.6	21.7	30.8
Associate’s degree	10.6	10.2	14.2	7.2
Bachelor’s Degree	19.1	19.2	21.3	16.8
Master’s degree professional or doctoral degree	9.4 3.7	8.6 4.1	9.5 3.2	10.0 4.0
**Annual household income**				
Less than $50,000	35.8	38.8	35.2	33.6
$50,000 **-** $100,000	35.5	33.9	36.8	34.4
More than $100,000	29.1	27.3	28.1	32.0
**Health status**				
Poor	3.2	3.3	3.2	3.2
Fair	16.2	18.4	15.0	15.2
Good	43.2	43.3	41.1	45.2
Very good	27.9	25.7	30.4	27.6
Excellent	9.5	9.4	10.3	8.8
**Pretest 30-day drinking frequency**				
Mean (SD)	8.7 (7.4)	8.6 (7.6)	8.9 (7.2)	8.7 (7.5)

**Fig 1:**
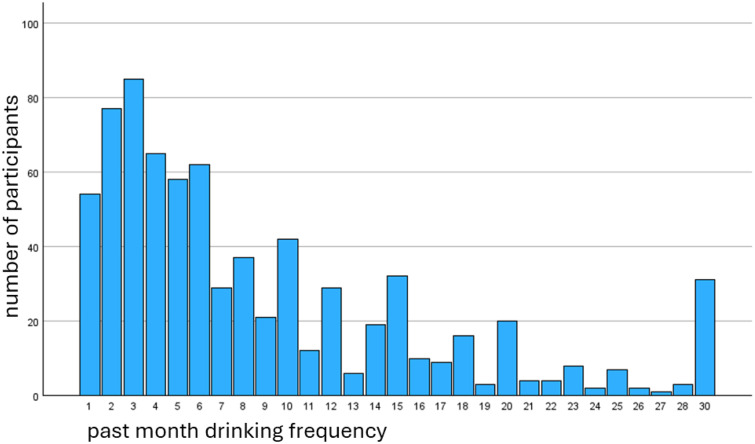
The distribution of participants’ drinking frequency over last 30 days.

All participants self-reported having consumed alcohol at least one day in the last 30 days. The average drinking frequency was 8.7 times (SD = 7.4) in the prior 30 days. Alcohol consumption frequency distribution is displayed in Fig 1 below.

Participants who viewed one of the health harm HWMs took a median time of 10.9 minutes to complete the entire survey. Responses ranged from 2 to 1296 characters in length, including spaces. Mean length was 118 characters (SD = 107) with a median of 93 characters. The average number of code labels applied to a response was 3 (SD = 2, range = 1–11; 11 occurring only once). This average number of code labels includes parent code and subcategory code labels.

## Results

### Research question 1 – types of responses

#### Non-substantive.

Sixteen (2.1%) responses included short, non-substantive phrases such as “no,” “nothing,” or “idk,” without additional development, explanation, or content. These responses were coded using the label *nonsubstantive* and were discrete without any additional code labels applied (i.e., no co-occurrences).

### Message impact framework constructs and sub-categories

#### Message reactions.

*Message reactions* was the most frequently used parent code, applied to 545 (72.9%) of the excerpts, meaning 545 participants included a message reaction in their responses to the HWM. We treated the subcategory code labels of *attention* and *cognitive elaboration* as types or gradations of message reactions. The code label *attention* was applied to excerpts that included simpler message reactions and restated the HWM content, such as “Drinking alcohol increases risk for breast cancer.” We applied the code *attention* to 165 excerpts (22.1%). The code label *cognitive elaboration* was applied to excerpts that expanded upon the HWM content, implying some level of attention or comprehension but also the ability to apply the content beyond reiteration of the message. Examples of *cognitive elaboration* excerpts are, “It made me think more deeply about the effects of alcohol” and “Makes me sad because I lost my ex due to a drinking problem…”. We applied the subcode *cognitive elaboration* to 148 excerpts (19.8%). Table 3 includes the code labels alongside exemplar responses.

**Table 3:** Code labels, exemplar responses, and percentage of codes applied in total and by health harm exposure (combined text-only HWM and picture-and-text HWM).

Code	Exemplar Response with Application of the Code (Participant Number)	Health Harm Exposure			
		Overall N = 748 (%)	Mortality condition n = 245 (%)	Mastectomy condition n = 253 (%)	Hair loss condition n = 250 (%)
**Nonsubstantive**	idk (Participant 623)	16 (2.1)	5 (2.0)	7 (2.8)	4 (1.6)
**Message Reactions**		545 (72.9)	176 (71.8)	190 (75.1)	179 (71.6)
Fear/Anxiety/Worry	Scared (Participant 643). It was scary when I saw it. It increases the risk of breast cance....I was scared (Participant 500).	157 (21.0)	49 (20.0)	57 (22.5)	51 (20.4)
Cognitive Elaboration	I know there are other serious conditions other than primary conditions that go along with drinking (Participant 731).	148 (19.8)	49 (20.0)	47 (18.6)	50 (20.0)
Attention	That not drinking can reduce the risk of contracting breast cancer. Breast cancer attacks cells for vital functions (Participant 57).	165 (22.1)	50 (20.4)	58 (22.9)	57 (22.8)
Hope	Drinking alcohol has scary consequences that are irreversible. It is scary to think that our bodies are effected by alcohol. However I am hopeful with the information being spread to help others understand the severity of drinking alcohol (also coded with Fear, Perceived Threat, Participant 103)	10 (1.3)	5 (2.0)	3 (1.2)	2 (0.8)
Sad	Sad (Participant 320)	47 (6.3)	16 (6.5)	16 (6.3)	15 (6.0)
Shock/Surprise	I thought it was alarming (Participant 477)	43 (5.7)	13 (5.3)	17 (6.7)	13 (5.2)
Seeking Information	How does drinking relate to breast cancer? How does not drinking reduce the risk of getting breast cancer? (also coded with Attention, Participant 662)	49 (6.6)	20 (8.2)	14 (5.5)	15 (6.0)
Survey Specific	1st graphic was the best one, simple & straight forward. Would like some numbers to show me how many women are affected by this (also coded with Needs Resources/ Credibility Participant 69)	91 (12.2)	22 (9.0)	36 (14.2)	33 (13.2)
**Attitudes and Beliefs**		435 (58.2)	146 (59.6)	147 (58.1)	142 (56.8)
First person and/or Perceived Efficacy	Cancer would be really bad and I would not want to lose my hair but at the same time I know I can quit drinking alcohol (Participant 703)	200 (26.7)	60 (24.5)	68 (26.9)	72(28.8)
Dismissal of current threat	Yes I know this. I’m not over doing my drinks so I’m ok (Participant 387)	62 (8.3)	20 (8.2)	26 (10.3)	16 (6.4)
Others – Perceived Threat	I didn’t know that. I wish more people knew that. Would people actually quit drinking if they knew that? Probably not... I wish alcohol was prohibited. (also coded New information, Participant 681)	149 (19.9)	50 (20.4)	59 (23.3)	40 (16.0)
Thinking about family/friends	I need to have my husband stop drinking, I work with alcoholics this message would be good for them (Participant 589)	73 (9.8)	26 (10.6)	22 (8.7)	25 (10.0)
New Information	I didn’t know it could cause breast cancer, so that was interesting to learn (Participant 26)	151 (20.2)	51 (20.8)	50 (19.8)	50 (20.0)
**Behavioral Intentions**		65 (8.7)	25 (10.2)	14 (5.5)	26 (10.4)
Reduce drinking	My reaction is that I should probably limit my alcohol consumption a bit more (Participant 169).	18 (2.4)	9 (3.7)	3 (1.2)	6 (2.4)
Stop drinking	I don’t really want to drink alcohol at all anymore(Participant 376).	48 (6.4)	17 (6.9)	11 (4.3)	20 (8.0)

* *Note: In the mortality condition, 123 participants were exposed to the text-only messages, while 122 participants were exposed to the picture-and-text messages. In the mastectomy condition, 132 participants were exposed to the text-only messages, while 121 participants were exposed to the picture-and-text messages. In the hair loss condition, 123 participants were exposed to the text-only messages, while 127 participants were exposed to the picture-and-text messages.*

This last example response also received the code label of *sad*. We delineated emotions using the subcategory code labels: *sad, hope, shock/surprise,* and *fear/anxiety/worry*. The code label *fear/anxiety/worry* was applied the most frequently with 157 responses (21.0%). The code labels *sad* and *shock/surprise* were applied to 47 (6.3%) and 43 (5.7%) of the responses, respectively. The code label of *hope* was applied to 10 responses (1.3%).

Ninety-one participants (12.2%) included comments about the survey. These responses included words or phrases such as “the survey was” or “it was” such as, “I thought it was very informative.” Twenty-four participants responded only about the survey, meaning their responses were only coded with the *survey specific* parent code. This also means that 67 (9.0%) participants responded specifically about the survey but also with additional content that fell within the Message Impact Framework construct categories.

### Attitudes and beliefs

The parent code label *attitudes and beliefs* applied to 435 responses (58.2%). The subcategory code labels for *attitudes and beliefs* evolved during analysis because this was one construct of the Message Impact Framework that was more nuanced or qualitatively subtle. We realized the risk of introducing bias and assumptions when evaluating what the perceptions of participants were when we only had the one written response without follow-up. For these reasons, we included two subcategories for *attitudes and beliefs* that we, as a research team, could objectively delineate between – *first person and/or perceived efficacy* and *perceived threat*.

*First person and/or perceived efficacy* labeled the I or me responses about the impact of the threat and their ability or lack thereof to address the threat. For instance, “The messages made me feel that maybe I could prevent myself or my family members from getting breast cancer by avoiding alcohol…” This code label was applied to 200 responses (26.7%) where participants acknowledged (or dismissed) the risk and its application to themselves. We used one subcategory label for the responses that dismissed the current threat. *Dismissal of current threat* was applied to 6*2* of the responses (8.3%) where participants acknowledged the threat in the HWM but also dismissed or deferred the threat, indicating that they were not at risk because of current drinking habits, age, or health status. An example of a response coded with this label is “*I don’t really drink alcohol much so those messages didn’t affect me. I understand the effects of alcohol but it can’t affect me if I don’t drink it hardly ever*.”

*Perceived threat* was applied to 149 responses (19.9%) where participants acknowledged the health harm for others, but not necessarily themselves. An example of a response coded with perceived threat is “*It makes me think how easily alcohol can affect someone’s mind and body. Drinking alcohol deteriorates your mind which is linked to your body and how it responds to the amount liquor*.” The subcategory of *thinking about family/friends* was applied to 73 responses (9.8%) to detail the number of participants who mentioned thinking of or telling their sister, mother, or friends about the relationship between alcohol and breast cancer risk. Responses receiving this code indicated participants’ application of how the health harm might impact their loved ones and/or an intention for future interpersonal communication about alcohol and increased risk for breast cancer.

Participants responded about how the HWMs presented information that was new to them. We applied the *new information* code label to 151 responses (20.2%). Responses receiving this code label included phrases such as “I didn’t know alcohol increases risk for breast cancer” or “*This is new information to me*,” and “*I did not know drinking could lead to breast cancer….This needs to be told to everyone*.” We applied the code *seeking information* to 49 responses (6.6%). Responses receiving this code label included phrases such as “*I want to know the source*” or “*what is the data behind this message*.”

### Behavioral intentions

The parent code *behavioral intentions* was applied to 65 excerpts (8.7%) including the subcategory code labels of *reduce drinking* (18 responses, 2.4%) and *stop drinking* (48 responses, 6.4%)

### Research question 2 – type of HWM (text-only or picture-and-text) and health harm and Written Response

There were no significant associations between the type of message viewed (text-only or picture-and-text) and the type of response. This included *message reactions* (*χ²* (1, N = 748) = 1.49, p = .22) and its subcode *attention* (*χ*² (1, N = 748) = 0.18, p = .67), *attitudes and beliefs* (*χ²* (1, N = 748) = 1.01, p = .32) and its subcode *first person and/or perceived efficacy* (*χ²* (1, N = 748) = 1.85, p = .17), *behavioral intentions* (*χ²* (1, N = 748) = 0.55, p = .46) and its subcode *stop drinking* (*χ²* (1, N = 748) = 2.46, p = .12).

No significant associations between the health harm viewed (mastectomy, hair loss from chemotherapy, and mortality) and type of response (text-only or picture-and-text) were identified. This included within *message reactions* (*χ²* (2, N = 748) = 0.97, p = .62) and its subcode *attention* (*χ²* (2, N = 748) = 0.58, p = .75), *attitudes and beliefs* (*χ²* (2, N = 748) = 0.40, p = .82) and its subcode *first person and/or perceived efficacy* (*χ²* (2, N = 748) = 2.46, p = .29), *behavioral intentions* (*χ²* (2, N = 748) = 4.81, p = .09) and its subcode *stop drinking* (*χ²* (2, N = 748) = 2.96, p = .23).

### Research question 3 – type of health harm, response, and demographic moderators

Age was the only variable tested that was associated with type of response. Although the age of participants was restricted between 20–29 years for our study, age was found to have a significant moderating effect on the hair loss from chemotherapy health harm HWMs (*β* = −0.04, *p* = .005) in terms of responses coded as *attitudes and beliefs*. Specifically, when participants were exposed to the hair loss from chemotherapy condition messages, an increase in age was associated with a lower likelihood of responding with attitude and belief related words or phrases.

## Discussion

Health warning messages about alcohol consumption and breast cancer risk may serve as a strategy for breast cancer prevention education. Our HWMs, regardless of whether they included pictures or were text-only and regardless of the health harm discussed, elicited responses that aligned with the constructs and corresponding variables of the Message Impact Framework. Our directed content analysis findings complement the results of our main experiment [40] and provide audience-driven insights into message design elements that may be needed for future public health messaging.

The Message Impact Framework constructs of *message reactions* and attention were identified most frequently in participants’ responses. This is important because successful HWM design must first elicit a reaction and capture recipients’ attention [30]. Because current alcohol product packaging in the US is not required to include information about cancer risk, there may be a missed opportunity to garner the attention needed to impact changes in behavior and public perception.

Participant responses indicated a number of emotional reactions to the HWMs. Health warnings that elicit emotional reactions by describing susceptibility to a health threat (i.e., threat appeals) can be effective at impacting health behaviors in a positive way (i.e., promoting health behavior), especially when the warnings include efficacy language [60]. We used efficacy language in our HWMs and found evidence of perceived efficacy as demonstrated by responses such as “That I am at extreme risk for breast cancer because of my family history as well as my drinking. These messages were motivational to quit drinking all together.” Message attributes that include components of self-efficacy may be beneficial to consider as US regulatory agencies examine strategies to increase awareness and educate the general public about alcohol use and cancer risk [61].

Responses corresponding to participants’ *attitudes and beliefs* were also identified. Some of the participants responded that they did not perceive a personal risk for breast cancer related to their alcohol consumption. These participants seemed to acknowledge the risk but responded that their own alcohol consumption behaviors would likely not increase their own risk for breast cancer given their age, health status, and low frequency of alcohol consumption. This *optimistic bias*, or the idea that there is a risk, but the risk is less likely to impact them personally, has been observed in other health behavior and public health arenas [62,63]. To address optimistic bias, future HWM design and public awareness campaigns could consider including language about: 1) how everyone who drinks alcohol may be increasing their risk for breast cancer, 2) how there is a dose dependency in the relationship, and/or 3) the sources of reliable information about the risk.

Exploring the Message Impact Framework constructs allowed for more detailed insight into how participants reacted to the HWMs. For example, 20% of participants chose to respond that the relationship between alcohol consumption and breast cancer risk was entirely new to them. This finding supports previous studies that have also found low public awareness about the relationship between alcohol consumption and breast cancer risk [13,15,17]. Acknowledging that the risk relationship introduced in a HWM may be new information to the target population could be an important first step in grounding communication to the audience’s current context and perspectives.

Additionally, although all health harms (mastectomy, hair loss related to chemotherapy, and mortality) in this study elicited responses related to the Message Impact Framework, we found that age had a moderating effect on responses about attitudes and beliefs in hair loss health harm. Specifically, younger participants were more likely to react to the hair loss from chemotherapy condition than older participants with reactions related to attitudes and beliefs. These findings suggest that demographic traits, such as age, could be considered in contextualizing HWM design, as values change with life experiences.

### Limitations

Within the survey, the open-ended response opportunity came after a series of demographic and other assessments. Participants could have been aware of the time spent on survey completion so far and typed less to finish sooner, thought less thoroughly about the HWM, or typed fewer words to spend less time. Likewise, the cross-sectional design did not allow for follow-up questions. Future studies examining the topic of alcohol consumption and breast cancer risk may consider additional in-depth qualitative inquiry to further assess health warning messaging attributes’ ability to change alcohol consumption behaviors. We used directed content analysis that allows for discussion and adjustments during the analysis process. We took many steps to reduce the risk of subjectivity, and our findings add to the growing body of science about HWMs in the alcohol and cancer space. Our sample did report a wide range of drinking frequencies. Future studies should further examine HWMs about alcohol and breast cancer risk among samples of nondrinkers and moderate to heavy drinkers. Lastly, although we found the moderating effect of age in this study, we restricted the age range to focus on the young adult group. Therefore, we should be cautious when interpreting age’s moderating effects to a broader age group. Future studies should further explore the effect of demographic characteristics, especially age, on participants’ reactions to messages, providing more practical guidance for health campaign message design.

## Conclusion

Our findings inform ongoing research about HWMs addressing the relationship between alcohol consumption and breast cancer risk. We qualitatively identified the constructs and corresponding variables of the Message Impact Framework within the open-ended responses that indicate the possible impact our HWMs could have on increasing awareness or potentially changing behaviors. Our analysis also identified a lack of awareness about alcohol consumption and breast cancer risk that indicates HWMs such as ours may be used to increase public knowledge and education. Future research should continue to consider the message attributes that are most effective for at-risk populations.

## References

[1] American Cancer Society. Breast Cancer Facts & Figures 2024-2025. 2024.

[2] Vineis P, Wild CP. Global cancer patterns: causes and prevention. Lancet. 2014;383(9916):549–57. doi: 10.1016/S0140-6736(13)62224-2 24351322

[3] Liu Y, Nguyen N, Colditz GA. Links between alcohol consumption and breast cancer: a look at the evidence. Womens Health (Lond). 2015;11(1):65–77. doi: 10.2217/whe.14.62 25581056 PMC4299758

[4] Anderson BO, Berdzuli N, Ilbawi A, Kestel D, Kluge HP, Krech R, et al. Health and cancer risks associated with low levels of alcohol consumption. Lancet Public Health. 2023;8(1):e6–7. doi: 10.1016/S2468-2667(22)00317-6 36603913 PMC9831798

[5] International Agency for Research on Cancer, International Agency for Research on Cancer, editors. Alcohol drinking: views and expert opinions: this publication represents the views and expert opinions of an IARC Working Group on the Evaluation of the Carcinogenic Risk of Chemicals to Humans which met in Lyon, 13 - 20 October 1987 [Internet]. Lyon: International Agency for Research on Cancer; 1988. https://publications.iarc.fr/Book-And-Report-Series/Iarc-Monographs-On-The-Identification-Of-Carcinogenic-Hazards-To-Humans/Alcohol-Drinking-1988

[6] Longnecker MP, Newcomb PA, Mittendorf R, Greenberg ER, Clapp RW, Bogdan GF, et al. Risk of breast cancer in relation to lifetime alcohol consumption. J Natl Cancer Inst. 1995;87(12):923–9. doi: 10.1093/jnci/87.12.923 7666482

[7] Ziembicki S, Zhu J, Tse E, Martin LJ, Minkin S, Boyd NF. The Association between Alcohol Consumption and Breast Density: A Systematic Review and Meta-analysis. Cancer Epidemiol Biomarkers Prev. 2017;26(2):170–8. doi: 10.1158/1055-9965.EPI-16-0522 27672053

[8] National Academies of Sciences, Engineering, and Medicine. Review of Evidence on Alcohol and Health. 2024. https://www.nationalacademies.org/our-work/review-of-evidence-on-alcohol-and-health#sectionSponsors

[9] Rumgay H, Murphy N, Ferrari P, Soerjomataram I. Alcohol and Cancer: Epidemiology and Biological Mechanisms. Nutrients. 2021;13(9):3173. doi: 10.3390/nu13093173 34579050 PMC8470184

[10] Colditz GA, Bohlke K, Berkey CS. Breast cancer risk accumulation starts early: prevention must also. Breast Cancer Res Treat. 2014;145(3):567–79. doi: 10.1007/s10549-014-2993-8 24820413 PMC4079839

[11] Shield KD, Soerjomataram I, Rehm J. Alcohol use and breast cancer: a critical review. Alcohol Clin Exp Res. 2016;40(6):1166–81. doi: 10.1111/acer.13071 27130687

[12] Siegel RL, Kratzer TB, Giaquinto AN, Sung H, Jemal A. Cancer statistics, 2025. CA Cancer J Clin. 2025;75(1):10–45. doi: 10.3322/caac.21871 39817679 PMC11745215

[13] Khushalani JS, Qin J, Ekwueme DU, White A. Awareness of breast cancer risk related to a positive family history and alcohol consumption among women aged 15–44 years in United States. Prev Med Rep. 2020;17:101029. doi: 10.1016/j.pmedr.2019.101029 31890475 PMC6926360

[14] Seidenberg AB, Wiseman KP, Eck RH, Blake KD, Platter HN, Klein WMP. Awareness of alcohol as a carcinogen and support for alcohol control policies. Am J Prev Med. 2022;62(2):174–82. doi: 10.1016/j.amepre.2021.07.005 34654593 PMC11922179

[15] Scheideler JK, Klein WMP. Awareness of the link between alcohol consumption and cancer across the world: a review. Cancer Epidemiol Biomarkers Prev. 2018;27(4):429–37. doi: 10.1158/1055-9965.EPI-17-0645 29615419

[16] Swahn MH, Martinez P, Balenger A, Luningham J, Seth G, Awan S, et al. Demographic disparities in the limited awareness of alcohol use as a breast cancer risk factor: empirical findings from a cross-sectional study of U.S. women. BMC Public Health. 2024;24(1):1076. doi: 10.1186/s12889-024-18565-z 38637773 PMC11025251

[17] American Institute for Cancer Research. 2019 AICR Cancer Risk Awareness Survey [Internet]. 2019 p. 23. https://www.aicr.org/assets/can-prevent/docs/2019-Survey.pdf

[18] GBD 2020 Alcohol Collaborators. Population-level risks of alcohol consumption by amount, geography, age, sex, and year: a systematic analysis for the Global Burden of Disease Study 2020. Lancet. 2022;400(10347):185–235. doi: 10.1016/S0140-6736(22)00847-9 35843246 PMC9289789

[19] Gallup Inc. U.S. Drinking Rate at New Low as Alcohol Concerns Surge. Gallup.com. https://news.gallup.com/poll/693362/drinking-rate-new-low-alcohol-concerns-surge.aspx. 2025. Accessed 2025 September 22.

[20] Substance Abuse and Mental Health Services Administration. Companion Infographic Report: Results from the 2021, 2022, and 2023 National Surveys on Drug Use and Health. 2024.

[21] Martinez P, Patterson D, Yu Y, Greenfield T, Kerr W. Fewer people drinking more: changes in alcohol use and demographic differences between 2019 and 2024. New Orleans, Louisiana; 2025.

[22] The US Surgeon General’s Advisory. Alcohol and Cancer Risk 2025. 2025;22.

[23] TTB A and TT and TB. Beverage Alcohol. https://www.ttb.gov/regulated-commodities/beverage-alcohol/distilled-spirits/ds-labeling-home/ds-health-warning. 2024. Accessed 2025 February 27.

[24] Zhao J, Stockwell T, Vallance K, Hobin E. The effects of alcohol warning labels on population alcohol consumption: an interrupted time series analysis of alcohol sales in Yukon, Canada. J Stud Alcohol Drugs. 2020;81(2):225–37. doi: 10.15288/jsad.2020.81.225 32359054

[25] Stockwell T, Solomon R, O’Brien P, Vallance K, Hobin E. Cancer Warning Labels on Alcohol Containers: A Consumer’s Right to Know, a Government’s Responsibility to Inform, and an Industry’s Power to Thwart. J Stud Alcohol Drugs. 2020;81(2):284–92. doi: 10.15288/jsad.2020.81.284 32359059

[26] Canada’s guidance on alcohol and health. https://www.ccsa.ca/canadas-guidance-alcohol-and-health. Accessed 2025 February 20.

[27] Office of the Attorney General I. S.I. No. 249/2023 - Public Health (Alcohol) (Labelling) Regulations 2023. Office of the Attorney General. 2023. https://www.irishstatutebook.ie/eli/2023/si/249/made/en/print

[28] Slattery C. How Ireland beat the odds to introduce cancer warning labels on alcohol. World Cancer Res. Fund. https://www.wcrf.org/about-us/news-and-blogs/how-ireland-beat-the-odds-to-introduce-cancer-warning-labels-on-alcohol/. 2024. Accessed 2025 February 20.

[29] Murphy M. Ireland delays cancer warning label for alcohol. Ir. Post. https://www.irishpost.com/news/ireland-delays-cancer-warning-label-for-alcohol-294341. Accessed 2025 September 20.

[30] Wogalter MS, Conzola VC, Smith-Jackson TL. Research-based guidelines for warning design and evaluation. Appl Ergon. 2002;33(3):219–30. doi: 10.1016/s0003-6870(02)00009-1 12164506

[31] Noar SM, Barker J, Bell T, Yzer M. Does perceived message effectiveness predict the actual effectiveness of tobacco education messages? a systematic review and meta-analysis. Health Commun. 2020;35(2):148–57. doi: 10.1080/10410236.2018.1547675 30482058 PMC6538475

[32] Hammond D. Health warning messages on tobacco products: a review. Tob Control. 2011;20(5):327–37. doi: 10.1136/tc.2010.037630 21606180

[33] Ma Z, Ma R. Designing cancer warning labels for alcoholic beverages: examining the impact of visual elements. Health Educ Behav. 2023;50(5):586–94. doi: 10.1177/10901981231166696 37131330

[34] Ma Z. “I Can See a Story from the Warning”: Understanding the role of perceived narrativity in pictorial warning labels. Health Commun. 2024;39(4):675–84. doi: 10.1080/10410236.2023.2181050 36803193

[35] Kokole D, Anderson P, Jané-Llopis E. Nature and Potential Impact of Alcohol Health Warning Labels: A Scoping Review. Nutrients. 2021;13(9):3065. doi: 10.3390/nu13093065 34578942 PMC8469468

[36] Ma Z, Haworth J, Hu J. Effects of narrative versus non-narrative pictorial warning labels on visual attention and alcohol-related cancer risk perceptions: An eye-tracking study. Addict Behav. 2025;162:108229. doi: 10.1016/j.addbeh.2024.108229 39671807 PMC11725444

[37] Grummon AH, Ruggles PR, Greenfield TK, Hall MG. Designing Effective Alcohol Warnings: Consumer Reactions to Icons and Health Topics. Am J Prev Med. 2023;64(2):157–66. doi: 10.1016/j.amepre.2022.09.006 37575887 PMC10421534

[38] Grummon AH, Lee CJY, D’Angelo Campos A, Whitesell C, Brewer NT, Lazard AJ, et al. Health harms that discourage alcohol consumption: a randomized experiment of warning messages. Addict Behav. 2024;159:108135. doi: 10.1016/j.addbeh.2024.108135 39191066 PMC11407683

[39] Grummon AH, Lee CJY, Campos AD, Lazard AJ, Brewer NT, Whitesell C, et al. New alcohol warnings outperform the current U.S. warning in a national survey experiment. J Stud Alcohol Drugs. 2025;10.15288/jsad.25-00226. doi: 10.15288/jsad.25-00226 40981644 PMC12574658

[40] Massey ZB, Anbari AB, Wang N, Adediran A, Lawrie LL, Martinez P, et al. Developing and testing health warnings about alcohol and risk for breast cancer: results from a national experiment with young adult women in the United States. Alcohol Clin Exp Res (Hoboken). 2025;49(3):665–77. doi: 10.1111/acer.70003 39985486 PMC12186991

[41] Bates S, Holmes J, Gavens L, de Matos EG, Li J, Ward B, et al. Awareness of alcohol as a risk factor for cancer is associated with public support for alcohol policies. BMC Public Health. 2018;18(1):688. doi: 10.1186/s12889-018-5581-8 29866082 PMC5987582

[42] Christensen ASP, Meyer MKH, Dalum P, Krarup AF. Can a mass media campaign raise awareness of alcohol as a risk factor for cancer and public support for alcohol related policies?. Prev Med. 2019;126:105722. doi: 10.1016/j.ypmed.2019.05.010 31125628

[43] Jones D, Moodie C, Purves RI, Fitzgerald N, Crockett R. The role of alcohol packaging as a health communications tool: An online cross-sectional survey and experiment with young adult drinkers in the United Kingdom. Drug Alcohol Rev. 2022;41(5):1206–15. doi: 10.1111/dar.13469 35385591

[44] Noar SM, Bell T, Kelley D, Barker J, Yzer M. Perceived message effectiveness measures in tobacco education campaigns: a systematic review. Commun Methods Meas. 2018;12(4):295–313. doi: 10.1080/19312458.2018.1483017 31428217 PMC6699787

[45] Haddock G, Zanna MP. On the use of open‐ended measures to assess attitudinal components. British J Social Psychol. 1998;37(2):129–49. doi: 10.1111/j.2044-8309.1998.tb01161.x

[46] Cacioppo JT. Social psychological procedures for cognitive response assessment: the thought listing technique. In: Merluzzi C, Glass C, Genest M, editors. Cogn Assess. New York: Guilford Press. 1981. p. 309–42.

[47] Geer JG. Do open-ended questions measure “Salient” Issues?. Public Opinion Quarterly. 1991;55(3):360. doi: 10.1086/269268

[48] Cacioppo JT, von Hippel W, Ernst JM. Mapping cognitive structures and processes through verbal content: the thought-listing technique. J Consult Clin Psychol. 1997;65(6):928–40. doi: 10.1037//0022-006x.65.6.928 9420354

[49] Noar SM, Francis DB, Bridges C, Sontag JM, Ribisl KM, Brewer NT. The impact of strengthening cigarette pack warnings: Systematic review of longitudinal observational studies. Soc Sci Med. 2016;164:118–29. doi: 10.1016/j.socscimed.2016.06.011 27423739 PMC5026824

[50] Elo S, Kyngäs H. The qualitative content analysis process. J Adv Nurs. 2008;62(1):107–15. doi: 10.1111/j.1365-2648.2007.04569.x 18352969

[51] Hsieh H-F, Shannon SE. Three approaches to qualitative content analysis. Qual Health Res. 2005;15(9):1277–88. doi: 10.1177/1049732305276687 16204405

[52] Assarroudi A, Heshmati Nabavi F, Armat MR, Ebadi A, Vaismoradi M. Directed qualitative content analysis: the description and elaboration of its underpinning methods and data analysis process. J Res Nurs. 2018;23(1):42–55. doi: 10.1177/1744987117741667 34394406 PMC7932246

[53] Neuendorf KA. Content analysis—a methodological primer for gender research. Sex Roles. 2010;64(3–4):276–89. doi: 10.1007/s11199-010-9893-0

[54] White AM. Gender differences in the epidemiology of alcohol use and related harms in the United States. Alcohol Res. 2020;40(2):01. doi: 10.35946/arcr.v40.2.01 33133878 PMC7590834

[55] U.S. Census Bureau QuickFacts: United States [Internet]. [cited 2025 Feb 20]. https://www.census.gov/quickfacts/fact/table/US/PST045223

[56] Tong A, Sainsbury P, Craig J. Consolidated criteria for reporting qualitative research (COREQ): a 32-item checklist for interviews and focus groups. Int J Qual Health Care. 2007;19(6):349–57. doi: 10.1093/intqhc/mzm042 17872937

[57] Duong HT, Massey ZB, Churchill V, Popova L. Are smokers scared by COVID-19 risk? How fear and comparative optimism influence smokers’ intentions to take measures to quit smoking. PLoS One. 2021;16(12):e0260478. doi: 10.1371/journal.pone.0260478 34874964 PMC8651098

[58] Massey ZB, Duong HT, Churchill V, Popova L. Examining reactions to smoking and COVID-19 risk messages: An experimental study with people who smoke. Int J Drug Policy. 2022;102:103607. doi: 10.1016/j.drugpo.2022.103607 35180555 PMC8801323

[59] Owusu D, So J, Popova L. Reactions to tobacco warning labels: predictors and outcomes of adaptive and maladaptive responses. Addict Res Theory. 2019;27(5):383–93. doi: 10.1080/16066359.2018.1531127 31511769 PMC6738943

[60] Witte K, Allen M. A meta-analysis of fear appeals: implications for effective public health campaigns. Health Educ Behav. 2000;27(5):591–615. doi: 10.1177/109019810002700506 11009129

[61] The Alcohol and Tobacco Tax and Trade Bureau (TTB). Labeling and advertising of wine, distilled spirits, and malt beverages with alcohol content, nutritional information, major food allergens, and ingredients. https://www.regulations.gov/docket/TTB-2024-0002. 2024.

[62] Weinstein ND. Optimistic biases about personal risks. Science. 1989;246(4935):1232–3. doi: 10.1126/science.2686031 2686031

[63] Bocquier A, Fressard L, Verger P, Legleye S, Peretti-Watel P. Alcohol and cancer: risk perception and risk denial beliefs among the French general population. Eur J Public Health. 2017;27(4):705–10. doi: 10.1093/eurpub/ckx024 28459975

